# Counts and cards – a novel way to detect typhoid infections in the ED

**DOI:** 10.1186/1865-1380-7-S1-P1

**Published:** 2014-07-25

**Authors:** Pragya Mallick, Aditya Maddali, Tausif Thangalvadi, Parivalavan Rajavelu

**Affiliations:** 1 Sundaram Medical Foundation Dr Rangarajan Memorial Hospital, Shanthi Colony, Anna Nagar, Chennai, India

## Objective

The diagnosis of typhoid fever is made by growth of the causative microorganism from culture of bone marrow aspirate or blood which is time dependent. In a busy ED like ours, typhoid fever is a frequent presentation. There is need for a rapid and reliable test made available at the bed side, to detect typhoid infections. The sensitivity and specificity of Enterocheck WB, a card test that detects IgM antibodies to salmonella typhi and low eosinophil counts were compared individually and together, with the gold standard of blood culture using BacT/Alert.

## Methods

Setting - Multi specialty community based teaching hospital in Chennai with about 1500 ED visits per month

Number of subjects – 95.

Study design - Retrospective study done from January 2012 for a period of 1 year.

## Results

For 4 or lesser days of fever, typhoid IgM had sensitivity of 66.67%, specificity of 40%. For 5 or greater days of fever, sensitivity was 75.61%, specificity 50%.

Eosinophil count was persistently low (0.09%) in all typhoid cases irrespective of day of presentation of fever.

Sensitivity of a low eosinophil count was 100%, specificity being 14.8%.

In all culture positive cases, sensitivity of IgM and low eosinophil counts together was 100%. For all culture negative cases, specificity was 92.3%. So both IgM and eosinophil count together have a high specificity as well as sensitivity.

## Limitations

This was a retrospective study. Prospective study is awaited to reconfirm the results.

## Conclusion

The typhoid IgM test can be performed at the bedside in the ED. Eosinophil count can be easily obtained from a CBC. In conjunction, they can accurately detect salmonella infections for early initiation of appropriate treatment in the ED itself, thereby saving time, money and precious hospital beds.

